# Yoga as salutogenic health promotion across displacement contexts: a culturally responsive public mental health synthesis

**DOI:** 10.3389/fpubh.2026.1815264

**Published:** 2026-07-30

**Authors:** Linda J. Douglas

**Affiliations:** Usher Institute, University of Edinburgh, Edinburgh, United Kingdom

**Keywords:** cultural responsiveness, forced displacement, psychosocial support, public mental health, salutogenesis

## Abstract

**Background:**

Global displacement is a major public mental health concern shaped by conflict, protracted geopolitical instability, disrupted social worlds, and structural inequities. Yoga is increasingly discussed within health promotion as a potentially accessible and adaptable practice. However, much of the existing literature has prioritized symptom-based outcomes and has rarely been situated within displacement-informed public mental health frameworks attentive to structural vulnerability, cultural heterogeneity, and ethical implementation conditions.

**Methods:**

This study undertakes a structured narrative synthesis of interdisciplinary literature spanning yoga-related practices, psychosocial wellbeing, salutogenic health promotion, public mental health, displacement, and culturally responsive care. Searches were conducted in MEDLINE via PubMed, Scopus, and PsycINFO, supplemented by backward and forward citation tracking of relevant reviews, humanitarian frameworks, and foundational theoretical works. Contemporary sources published between 2000 and 2025 were prioritized for literature addressing yoga, psychosocial wellbeing and public mental health. Seminal theoretical, methodological and policy sources published before 2000 were purposefully retained where they provided essential conceptual or methodological foundations for the synthesis. A final analytical corpus of 32 sources informed the synthesis, comprising empirical studies, systematic reviews, theoretical texts, and policy documents directly relevant to the conceptual framework. Additional literature informed background scoping and conceptual development but was not incorporated into the final analytical corpus. Yoga is conceptualized here as a heterogeneous practice category comprising contextually adapted combinations of movement, breath regulation, attentional practices, and optional contemplative elements, rather than as a singular intervention model. Sources were examined using iterative thematic analysis and conceptual mapping to identify explanatory mechanisms, contextual moderators, and ethical boundary conditions relevant to displacement contexts.

**Results:**

Three interrelated salutogenic pathways are identified: embodied self-regulation and perceived manageability under chronic stress; coherence, meaning, and purpose in disrupted lifeworlds; and social connectedness through community-based participation. These pathways are conceptualized as context-dependent processes shaped by cultural congruence, structural conditions, delivery modalities, safeguarding arrangements, and participant-defined interpretations of practice.

**Conclusion:**

Situating yoga within a salutogenic and culturally responsive public mental health framework clarifies how it may be interpreted as a preventive and community-oriented psychosocial resource within displacement contexts. The synthesis foregrounds ethical boundary conditions, including risks of cultural essentialism, depoliticization, individualizing structurally produced distress, and inadvertent harm. The framework is theory-generating and intended to inform empirically grounded research, context-sensitive implementation, and policy deliberation, rather than to assert generalized effectiveness.

## 1 Introduction

Global displacement constitutes a sustained public health and social policy concern affecting unprecedented numbers of people worldwide (1). Psychosocial wellbeing among displacement-affected populations is shaped not only by exposure to violence and loss, but also by protracted uncertainty, disrupted livelihoods, constrained rights, and the intersection of legal precarity with social exclusion. In many contexts, these conditions are embedded within geopolitical instability and long-term restrictions on mobility, work, education, family life, and access to services, with cumulative consequences for mental health and social functioning. Public mental health scholarship increasingly recognizes that individualized clinical models may be insufficient for addressing such layered determinants, particularly where service access is constrained and wellbeing is shaped by social determinants and relational worlds.

In displacement-affected settings, psychosocial distress is frequently produced and sustained through the interaction of structural constraint and everyday adversity, rather than through discrete traumatic exposures alone. Public mental health approaches therefore emphasize prevention, community platforms, and the social production of vulnerability, including the ways in which legal status, housing insecurity, restricted labor access, poverty, and discrimination shape wellbeing over time (2, 3). Within humanitarian governance, psychosocial strategies are commonly conceptualized as layered and interdependent, such that non-specialist and community-oriented practices are relevant only when situated within broader ecologies of safety, protection, and basic services (4). This socio-structural framing is analytically significant for yoga: if introduced as an individual coping technique without attention to rights-based support, yoga may inadvertently contribute to depoliticised understandings of distress.

Accordingly, conceptual clarity is required regarding how yoga may be positioned within prevention-oriented public mental health paradigms that remain attentive to structural determinants and do not displace responsibility from institutions to individuals.

Yoga, however, does not denote a unitary practice. Contemporary usages encompass diverse forms, including philosophical traditions, modern postural systems, therapeutic adaptations, secular wellbeing programmes, and commercialized transnational variants (5–7). Conceptual precision is therefore necessary when considering yoga within public health settings. Systematic reviews and meta-analyses report associations between yoga-based interventions and reductions in anxiety, depression, and perceived stress across heterogeneous populations. Nonetheless, much of this evidence remains situated within therapeutic paradigms emphasizing symptom outcomes and individual-level mechanisms. Comparatively limited attention has been paid to how yoga may be conceptualized within displacement-informed public mental health frameworks that foreground prevention, equity, structural determinants, and the cultural mediation of embodied practices.

Salutogenesis provides a theoretically robust anchor for addressing these questions. Rather than centring pathology, salutogenic approaches foreground resources, capacities, and conditions that support movement toward health, particularly sense of coherence, manageability, and meaningfulness. In displacement contexts, salutogenesis is pertinent because chronic uncertainty may erode coherence and diminish perceived manageability, thereby amplifying distress even where clinical disorder cannot be straightforwardly inferred. From this standpoint, yoga may be conceptualized not primarily as treatment, but as a potential health-promoting practice through which psychosocial resources may be cultivated in context.

However, such conceptualization requires careful contextualization. Displacement settings are not culturally homogeneous, and health practices are mediated by locally situated understandings of body, distress, care, gender, modesty, religion, and moral authority. Moreover, yoga's global circulation has produced multiple framings, including secular exercise, spiritual discipline, therapeutic practice, cultural tradition, and commercialized wellness activity, each of which may carry differing implications for acceptability and meaning. There is therefore a need for conceptual clarity regarding how yoga is theorized within public health, which mechanisms may be relevant across diverse displacement contexts, and which ethical risks may arise when practices travel globally and are introduced within structurally constrained and politically sensitive settings.

This article develops a structured narrative synthesis situated at the intersection of salutogenic health promotion, culturally responsive public mental health, and displacement-related structural vulnerability. The aim is to clarify how yoga may be interpreted within preventive public mental health paradigms, while identifying contextual moderators and ethical boundary conditions that shape feasibility, acceptability, and risk.

## 2 Conceptual and theoretical orientation

### 2.1 Salutogenesis and health-promoting resources

Antonovsky's salutogenic model conceptualizes health not as the absence of disease but as movement along a continuum shaped by access to general resistance resources and by the development of a sense of coherence through which life is experienced as comprehensible, manageable, and meaningful (8, 9). In public mental health, this orientation complements social determinants frameworks and capability-informed approaches that emphasize agency, equity, and structural conditions (10, 11). Within displacement contexts characterized by protracted uncertainty and constrained autonomy, salutogenesis provides a theoretically robust vocabulary for analyzing how psychosocial resources may be cultivated without collapsing such resources into clinical treatment paradigms.

### 2.2 Public mental health, prevention, and equity

Public mental health prioritizes prevention, community-based support, and equity-oriented governance (2, 12). This orientation is consistent with the principles of health promotion articulated in the Ottawa Charter, which emphasizes enabling people to increase control over their health and wellbeing (13). Humanitarian frameworks similarly emphasize layered systems of support in which psychosocial strategies are embedded within safe, rights-based infrastructures rather than delivered as isolated solutions (4). Such approaches caution against individualizing distress that is structurally produced by legal precarity, poverty, discrimination, conflict exposure, or constrained access to services (3, 14). Any conceptualization of yoga within displacement settings must therefore be situated within prevention-oriented and structurally attentive frameworks. This is consistent with sociological accounts of mental health that locate distress within wider social, institutional, and structural conditions, rather than solely within individual pathology (15).

### 2.3 Culture, religion, health, and displacement

Cultural responsiveness requires contextualization rather than translation alone (16, 17). Displacement-affected populations are heterogeneous in religious traditions, gender norms, embodied practices, explanatory models of distress, and moral worlds. Yoga's global circulation has produced multiple interpretations, including secular, spiritual, therapeutic, cultural, and commercial framings (5). Its acceptability and meaning are therefore mediated by local epistemologies of body, care, wellbeing, and moral authority.

Cultural responsiveness functions here as an analytic and ethical condition rather than an optional adaptation. This is particularly salient in displacement contexts characterized by religious plurality, differing bodily norms, and diverse understandings of health and care. In such settings, yoga may be understood as exercise, spiritual practice, therapeutic activity, culturally unfamiliar discipline, or valued tradition. Its acceptability and meaning should therefore be treated as contextually and locally mediated rather than assumed in advance.

## 3 Materials and methods

### 3.1 Design

This study adopts a conceptual analysis supported by structured narrative synthesis. As the article does not report a clinical trial, observational epidemiological study, or primary qualitative interview or focus-group study, CONSORT, STROBE, and COREQ reporting frameworks are not directly applicable. The aim is not effect estimation but theoretical clarification and model development. Narrative synthesis is appropriate where evidence is heterogeneous in design, modality, terminology, and outcome focus (18, 19). The analysis is based exclusively on published scholarly literature and publicly available policy or guidance documents; no primary empirical data were collected or analyzed.

### 3.2 Information sources and search strategy

Literature searches were undertaken in MEDLINE via PubMed, Scopus, and PsycINFO between January and February 2026. Searches prioritized contemporary sources published between January 2000 and December 2025, reflecting the expansion of contemporary yoga-health research alongside growing scholarship in public mental health and forced displacement. Seminal theoretical, methodological and policy sources published before 2000 were purposefully retained where they provided essential conceptual or methodological foundations for the synthesis. Searches were supplemented through backward and forward citation tracking of relevant systematic reviews, foundational theoretical texts, and key humanitarian guidance frameworks.

Search terms were organized across four concept domains to support conceptual breadth rather than exhaustive systematic retrieval: (i) yoga-related practices, including yoga, pranayama, breathwork, and mind-body practice; (ii) public mental health and health promotion, including prevention, wellbeing, psychosocial support, and social determinants; (iii) displacement-related contexts and populations, including refugee, asylum seeker, forced migration, and internally displaced persons; and (iv) salutogenesis and culturally responsive care, including sense of coherence, resistance resources, cultural adaptation, religion, and health.

Boolean combinations and database-adapted syntax were used where appropriate. Given disciplinary variation in terminology, citation tracking was employed to identify field-defining publications relevant to embodied regulation, meaning-making, and community participation (18, 19).

### 3.3 . Eligibility logic and corpus characteristics

The final analytical corpus comprised 32 published sources, including empirical studies, systematic reviews and meta-analyses, theoretical or historical scholarship, and policy or guidance documents directly relevant to the conceptual framework. Inclusion was guided by conceptual relevance to psychosocial wellbeing, salutogenic resources, yoga-related practices, displacement contexts, and culturally responsive implementation. Additional literature informed background scoping and conceptual development but was not incorporated into the final analytical corpus. As the objective was conceptual integration rather than exhaustive effect estimation, the corpus was assembled through theoretically informed purposive selection consistent with structured narrative synthesis (18, 19). The 32 sources included in the analytical corpus are presented in Supplementary Table S1.

Relevant materials included peer-reviewed intervention studies and systematic reviews addressing psychosocial outcomes associated with yoga, including anxiety, depressive symptoms, perceived stress, wellbeing, and functional indicators. The corpus also included displacement and refugee mental health scholarship relevant to chronic uncertainty, daily stressors, social exclusion, and structural vulnerability. Foundational theoretical texts were retained where central to conceptual definition, including salutogenesis and explanatory models of distress, while humanitarian governance documents were included where they provided widely used public mental health frameworks for implementation contexts (4, 12). Studies were excluded where yoga practices were insufficiently described to permit meaningful interpretation, where outcomes were not relevant to psychosocial wellbeing, or where relevance to displacement-informed public mental health was minimal.

### 3.4 Analytic procedure

Themes were refined through iterative comparison across disciplinary literatures using principles consistent with thematic analysis and conceptual synthesis (20, 21). Conceptual mapping was then employed to integrate themes into a coherent explanatory framework specifying salutogenic pathways, contextual moderators, and ethical boundary conditions.

Analytic integration proceeded in three stages. First, sources were read in full to identify recurring explanatory claims concerning embodied regulation, meaning-making, social participation, implementation conditions, and reported limitations. Secondly, these claims were compared across public mental health, psychosocial, humanitarian, displacement, and yoga-related literatures in order to identify convergences, tensions, and differing disciplinary emphases. Thirdly, conceptual mapping was used to specify relationships between salutogenic constructs, including comprehensibility, manageability, meaningfulness, and resistance resources (9), and the proposed pathways, while also delineating moderators affecting feasibility, acceptability, and ethical implementation.

Analytic refinement continued until additional sources were contributing conceptual repetition rather than materially distinct explanatory insights. Mixed, null, or cautionary findings, including limited acceptability, implementation barriers, adverse responses, or context-dependent outcomes, were retained to strengthen boundary conditions and to avoid overstatement of potential benefit.

### 3.5 Ethics statement

This article is a conceptual analysis based on structured narrative synthesis of previously published literature and publicly available policy and guidance documents. No primary data involving human participants were collected, and no identifiable human data are presented. As no primary research involving human participants was conducted, this study did not require review by a research ethics committee.

## 4 Findings: a salutogenic conceptualization of yoga in displacement contexts

This synthesis develops a salutogenic model through which yoga may be interpreted as a culturally responsive form of health promotion across displacement-affected contexts. Rather than treating yoga as a uniform intervention, the synthesis identifies three interrelated pathways through which embodied practice may plausibly contribute to psychosocial wellbeing under conditions of chronic uncertainty, constrained resources, and disrupted social worlds. These pathways are not posited as universal effects; instead, they are conceptualized as contingent processes shaped by cultural acceptability, structural conditions, and modes of implementation.

The three pathways identified through the synthesis are summarized in Table 1. The table makes explicit the relationship between evidence domains, salutogenic mechanisms, contextual moderators, ethical boundary conditions, and future research implications.

**Table 1:** Salutogenic pathways, evidence domains, contextual moderators, and ethical boundary conditions.

Salutogenic pathway	Main evidence domain	Proposed public mental health relevance	Contextual moderators	Ethical boundary conditions	Implications for research and implementation
Embodied self-regulation and perceived manageability under chronic stress	Yoga mental health literature; breath regulation and autonomic modulation; trauma-informed practice; displacement-related chronic stress scholarship.	Yoga may support perceived manageability through breath awareness, attentional anchoring, pacing, postural stability, and restoration of bodily predictability. It is conceptualized as a psychosocial resource rather than a clinical treatment.	Safety; bodily autonomy; trauma history; gender norms; modesty; disability; available space; privacy; facilitator training; continuity of delivery.	Participation must be voluntary, paced, non-coercive, and trauma-informed. Practice should not be framed as a substitute for protection, mental health services, legal support, or material assistance.	Future studies should examine acceptability, adverse responses, dosage, facilitator training, and participant-defined indicators of manageability, rather than relying only on symptom-reduction measures.
Coherence, meaning, and purpose in disrupted lifeworlds	Salutogenic theory; displacement scholarship on uncertainty, disrupted identity, and social worlds; cultural and religious understandings of health and care.	Structured and repeatable practice may contribute to rhythm, predictability, and perceived coherence in contexts marked by legal precarity, waiting, fragmented routines, and uncertainty.	Religious plurality; local epistemologies of body and care; participant interpretations of yoga; trust in facilitators; community infrastructure; compatibility with existing moral or spiritual frameworks.	Yoga should not be presumed meaningful, acceptable, or culturally neutral. Meaning must be locally negotiated, with scope for secular, movement-based, breath-based, or relaxation-focused adaptation where appropriate.	Future qualitative and mixed-methods research should examine how participants interpret yoga, for example as exercise, spiritual discipline, therapeutic practice, culturally unfamiliar activity, or community participation.
Social connectedness through community-based participation	Public mental health literature on social participation and protective determinants; humanitarian layered-support models; community-based psychosocial support.	Group-based yoga may offer a low-threshold form of shared participation, relational recognition, and non-clinical community routine when embedded in safe and inclusive settings.	Cost; location; language; gendered access; disability inclusion; childcare; facilitator accountability; community trust; organizational safeguarding; referral pathways.	Programmes may reproduce exclusion if they assume particular bodily capacities, familiarity with yoga, or access to private space. Social benefits are contingent rather than automatic.	Future implementation studies should compare individual and group formats, camp-based and urban settings, and the role of language mediation, safeguarding, accessibility, and organizational accountability.

### 4.1 Embodied self-regulation and perceived manageability under chronic stress

Chronic stress is a defining feature of protracted displacement, particularly where insecurity and uncertainty are ongoing rather than episodic (3, 14). Psychophysiological literature suggests plausible associations between breath regulation and autonomic modulation, indicating potential relevance to stress-related outcomes (22). Within a salutogenic register, yoga is conceptualized not as therapy, but as a potential resource for perceived manageability through embodied self-regulation, including breath awareness, postural stability, attentional anchoring, pacing, and the restoration of bodily predictability. These capacities are understood as psychosocial resources that may support coping, participation, and everyday functioning.

However, this pathway is contingent upon careful adaptation. Intensive or prescriptive forms of practice may be unsuitable, particularly where bodily autonomy has been compromised. Trauma-informed approaches emphasizing choice, pacing, and safety may be necessary to reduce risks of distress amplification (23, 24). The feasibility of embodied regulation is also shaped by culturally situated norms of bodily comportment, modesty, gendered participation, disability, privacy, and available space. Within layered support frameworks, embodied practices should therefore be framed as adjunctive resources rather than as substitutes for protection, mental health services, legal support, or material assistance, particularly where distress is linked to ongoing threat, discrimination, or material deprivation (4).

### 4.2 Coherence, meaning, and purpose in disrupted lifeworlds

This pathway is anchored explicitly in Antonovsky's concept of sense of coherence, which proposes that health is supported when individuals experience life as comprehensible, manageable, and meaningful (9). In displacement contexts, prolonged uncertainty, disrupted social worlds, constrained autonomy, and fragmented future horizons may undermine all three dimensions of sense of coherence. Within this salutogenic framework, structured and repeatable embodied practices may contribute to psychosocial wellbeing by supporting experiences of coherence, meaning, and perceived manageability amid conditions of instability and disruption.

Displacement disrupts temporal continuity, identity, social belonging, and future orientation. Protracted uncertainty regarding legal status, livelihood, mobility, and family life may erode perceived comprehensibility and meaningfulness. Structured and repeatable embodied practice may introduce rhythm and predictability into daily life. Within Antonovsky's salutogenic framework, such regularity may support the development or maintenance of sense of coherence through enhanced comprehensibility, perceived manageability, and meaningfulness, particularly where everyday life is otherwise fragmented by instability and constraint.

Meaning, however, is not inherent to yoga practice. It emerges through the ways practice is embedded in routine, social relations, religious or moral frameworks, and culturally mediated understandings of the body and care. Yoga may be regarded by some participants as compatible with existing spiritual or wellbeing practices, while others may experience the term itself as culturally unfamiliar, morally incongruent, or religiously inappropriate. In such contexts, discrete components such as breath regulation, stretching, relaxation, or movement-based practice may be more acceptable than presenting yoga as a singular practice category.

In salutogenic terms, coherence is not reducible to individual cognition; it is produced through social continuity, recognizable roles, credible future horizons, and trusted community infrastructures. A structured practice may contribute to coherence insofar as it offers a repeatable rhythm that can be integrated into daily routines and shared across social relationships. Conversely, coherence may be undermined where yoga is interpreted as culturally alien, religiously incompatible, or associated with externally imposed health agendas. The synthesis therefore treats meaning as emergent, negotiated, and ethically sensitive rather than assumed (16, 25).

### 4.3 Social connectedness through community-based participation

Social support and participation are recognized protective determinants of mental health and wellbeing (2, 26). Group-based yoga formats may foster participation, relational recognition, and accessible community routines where implemented inclusively and safely. Within a salutogenic framing, yoga may function as a relational practice that supports social connection through shared embodied participation.

However, social benefits are not automatic. Exclusionary dynamics may arise if programmes are inaccessible because of cost, location, language, gender norms, disability, childcare responsibilities, or implicit expectations about bodily capacity and familiarity with yoga. If yoga is framed exclusively as an individual coping strategy, attention may also be diverted from structural drivers of distress, including legal precarity, discrimination, poverty, and restricted access to services.

From a public mental health perspective, social connectedness operates both as a protective factor and as a mechanism through which prevention becomes practicable. Community-based yoga may therefore be interpreted as a platform for social participation, particularly where it is low-threshold, culturally respectful, and facilitated with sensitivity to gender norms, safeguarding, and organizational accountability. The framework consequently conceptualizes social connectedness as a contingent pathway whose benefits depend on inclusive design, trusted facilitation, and wider social conditions that allow participation and trust to be built and sustained.

### 4.4 Cultural responsiveness, ethical considerations, and structural conditions

Yoga cannot be presumed universally acceptable. Displacement contexts are heterogeneous in cultural norms, religious traditions, gender relations, and embodied practices of care. Ethical implementation requires attention to language, clothing norms, physical proximity, facilitation style, modesty, the social positioning of instructors, and participants' own interpretations of practice. Instructor accountability is likely to depend upon trauma-informed and culturally responsive training, clear professional boundaries, appropriate supervision, referral pathways where distress emerges, and accessible participant feedback processes. These overarching ethical and structural considerations are summarized in Table 2.

**Table 2:** Overarching ethical and structural considerations relevant to all salutogenic pathways.

Consideration	Description
Main evidence domain	Humanitarian guidance; public mental health equity frameworks; cultural responsiveness literature; social determinants of mental health
Public mental health relevance	Yoga may be considered a possible adjunctive resource within broader psychosocial and rights-based systems, rather than a stand-alone solution
Contextual moderators	Legal status; housing; livelihood insecurity; discrimination; access to services; protection risks; institutional capacity; continuity of provision
Ethical considerations	Risks include cultural essentialism, depoliticization, individualizing structurally produced distress, and inadvertent harm. Safeguarding, voluntariness, and referral capacity are essential
Research implications	Future research should situate yoga within layered systems of support and report implementation conditions transparently, including safeguarding, referral mechanisms, instructor training, adaptation processes, and participant feedback

The synthesis highlights the importance of avoiding homogenizing assumptions. Displaced populations should not be treated as a singular cultural category, nor should yoga be positioned as culturally neutral. Risks arise where individual practices are promoted without adequate attention to structural determinants of distress, or where “culture” is treated as static rather than socially dynamic. Any potential role for yoga in displacement contexts is therefore contingent upon cultural congruence, voluntariness, safeguarding, instructor accountability, and integration within broader rights-based systems.

These ethical boundary conditions are not ancillary; they are constitutive of whether yoga can be considered a public mental health resource at all. This conceptualization remains consistent with layered-support models that situate psychosocial practices within broader systems of care and governance (4, 12).

## 5 Discussion

A substantial proportion of yoga research has been framed within individual therapeutic paradigms, with symptom-based outcomes often prioritized. Meta-analyses have reported associations between yoga and reductions in anxiety, depression, and perceived stress across heterogeneous populations (27–29). However, variation in yoga modality, intensity, instructor training, delivery context, and outcome measurement complicates interpretation, while evidence specific to displacement-affected populations remains comparatively limited. The present synthesis does not seek to establish clinical efficacy. Instead, it reinterprets existing evidence through preventive, salutogenic, and structurally attentive public mental health frameworks, asking how yoga may support psychosocial resources, coherence, and wellbeing under conditions of chronic uncertainty, without detaching individual practice from the social and political determinants of distress.

### 5.1 Positioning within yoga mental health literature

Rather than asserting clinical efficacy, this analysis offers a conceptual reframing of yoga-related mental health evidence through a salutogenic lens. It shifts the analytic emphasis from symptom reduction alone toward the cultivation of psychosocial resources, perceived manageability, and sense of coherence in contexts characterized by chronic uncertainty. This framing provides a structured basis for interpreting variability across yoga modalities and implementation conditions without implying universal or generalizable effects.

A key contribution of this synthesis is to specify how findings reported in yoga mental health literature, including meta-analytic evidence, may be interpreted without converting heterogeneous or correlational evidence into broad claims of effectiveness. Where studies report symptom reduction, the present framework understands these outcomes as potentially reflecting changes in embodied self-regulation, perceived manageability, or coherence, while recognizing that such changes may not translate into sustainable wellbeing in the absence of social and structural support. Heterogeneity in yoga modalities, populations, settings, and delivery conditions further suggests that “yoga” functions as an umbrella category rather than a singular intervention. Conceptual clarification is therefore necessary to distinguish between possible mechanisms, implementation conditions, and contextual moderators.

The wider yoga evidence base is also constrained in places by small samples, short follow-up periods, weak comparators, and variable risk of bias, warranting cautious interpretation (28, 29). By delineating salutogenic pathways and ethical boundary conditions, this manuscript provides an analytic structure through which future empirical work may differentiate between modality-specific effects, context-sensitive outcomes, and the social conditions that shape whether yoga-based practices are acceptable, feasible, or beneficial in displacement settings.

### 5.2 Relevance to the displacement literature

The framework aligns with scholarship emphasizing daily stressors, legal precarity, and structural determinants as central to mental health outcomes in displacement contexts (3, 14). It also complements humanitarian layered-support models that conceptualize psychosocial strategies as embedded within safe systems of care rather than as isolated solutions (4). Within this perspective, yoga is interpreted as one potential resource among multiple supports rather than as a substitute for rights-based protection or material assistance.

Displacement scholarship has repeatedly demonstrated that post-displacement conditions and daily stressors may account for substantial variance in psychosocial wellbeing, often exceeding the explanatory capacity of trauma exposure alone (3). The present framework is aligned with that emphasis by treating yoga as a potential resource only insofar as it interfaces with the material and relational conditions of everyday life. Access to safe space, continuity of delivery, trust in facilitators, safeguarding capacity, and referral infrastructure are therefore not peripheral considerations, but determinants of whether implementation is ethically defensible. This emphasis strengthens alignment with public mental health paradigms that foreground equity and prevention, particularly in contexts where service provision is limited and psychosocial support must be integrated into community infrastructures (2, 12). Epidemiological and meta-analytic evidence similarly indicates the relevance of both pre-displacement exposure and post-displacement conditions in shaping mental health outcomes among refugees and displaced populations (30, 31).

### 5.3 Ethical boundary conditions and implementation considerations

The ethical credibility of implementation is likely to depend upon voluntariness, cultural responsiveness, safeguarding, instructor accountability, and integration within wider systems of support, so that structurally produced distress is not reframed as an individual deficit requiring self-management. Cultural essentialism is a parallel risk when displaced populations are treated as homogeneous or when yoga is presented as culturally neutral. Accordingly, any potential role for yoga should be understood as conditional rather than universal, dependent on context-sensitive adaptation, trauma-informed facilitation, and clear referral pathways where distress is elicited through embodied practice.

Ethical implementation also requires that yoga is not positioned as a solution to displacement-related distress, nor as a substitute for protection, legal support, or material assistance. Its role is therefore best conceptualized as bounded and adjunctive: a community-oriented wellbeing practice that may have value within rights-based and psychosocial systems, provided participation remains voluntary, culturally meaningful, and responsive to local understandings of body, distress, and healing. This is particularly important in displacement contexts where power asymmetries may be intensified. Conceptually, the framework makes explicit that proposed mechanisms of benefit cannot be separated from the social, cultural, and governance conditions under which practice is offered, interpreted, and received. Responsible public mental health implementation must therefore remain structurally attentive, rather than relocating responsibility for wellbeing onto displaced individuals themselves.

### 5.4 Limitations

This study employs structured narrative synthesis rather than systematic review methodology. It is theory-generating and does not claim exhaustive evidence retrieval, effect estimation, or generalizable effectiveness. As such, the analysis is not designed to establish the efficacy of yoga-based interventions, but to clarify conceptual relationships and identify theoretically plausible pathways for future investigation. The corpus was assembled through theoretically informed purposive selection across heterogeneous literatures, and therefore relevant studies may not have been captured comprehensively. This approach enabled engagement with diverse bodies of evidence, including public mental health, salutogenesis, displacement, trauma-informed practice, and yoga-related research, but it also limits claims regarding completeness and reproducibility of study identification. The framework should therefore be interpreted as an analytic model for conceptual clarification and future empirical testing, rather than as providing definitive conclusions regarding intervention effectiveness.

### 5.5 Future research

Future mixed-methods and longitudinal research across diverse displacement settings should disaggregate outcomes by gender, age, disability, legal status, and duration of displacement, as these factors may significantly shape feasibility, engagement, and potential benefit. Such differentiation is important for avoiding homogenized accounts of displaced populations and for identifying for whom, under what conditions, and through which mechanisms yoga-based approaches may be acceptable or beneficial.

Comparative research across camp-based and urban displacement environments may clarify how structural determinants, including legal status and livelihood insecurity, moderate potential effects. Transparent reporting of implementation conditions, instructor training, safeguarding procedures, and referral mechanisms would strengthen comparability across studies and enable more robust assessment of transferability across contexts.

Future research may also benefit from outcome conceptualization that extends beyond symptom metrics to include indicators more congruent with salutogenesis, such as perceived coherence, social participation, and functional manageability in daily life. Where empirical designs are feasible, implementation studies could examine how delivery characteristics, including group vs. individual formats, facilitator training, language mediation, and adaptation processes, interact with structural conditions, such as settlement type, service access, and legal constraints, to shape engagement and acceptability. Comparative qualitative work could illuminate local interpretive frames through which yoga is understood, for example as exercise, spiritual discipline, therapeutic practice, or culturally unfamiliar activity, thereby clarifying mechanisms of meaning-making and reasons for non-participation. Recovery-oriented constructs such as psychological detachment, relaxation, mastery, and control may also assist the operationalization of salutogenic outcomes where they are adapted cautiously to displacement contexts (32).

Such work would strengthen the evidence base while avoiding reductive generalization, and would support ethically responsible translation into policy and practice. It would also help to ensure that future research remains attentive not only to effectiveness, but also to cultural meaning, equity, implementation ethics, and the structural conditions that shape wellbeing in displacement contexts.

## 6 Conclusion

This conceptual analysis has situated yoga within a salutogenic and culturally responsive public mental health framework relevant to displacement contexts characterized by chronic uncertainty, structural inequity, and disrupted social worlds. Rather than positioning yoga as a clinical intervention or substitute for structural support, the article has identified three context-dependent salutogenic pathways through which embodied practice may support wellbeing: embodied self-regulation and perceived manageability under chronic stress; coherence, meaning, and purpose in disrupted lifeworlds; and social connectedness through community-based participation.

These pathways are not universal effects but contingent processes shaped by cultural congruence, implementation conditions, safeguarding practices, and broader governance environments. Cultural essentialism and depoliticization represent identifiable risks if yoga is framed as culturally neutral, or if individual coping practices are detached from the structural determinants of distress. The framework therefore emphasizes ethical boundary conditions, including voluntariness, cultural responsiveness, instructor accountability, trauma-informed facilitation, and integration within layered psychosocial and rights-based systems.

By reframing existing yoga and mental health literature through salutogenic and displacement-informed public mental health perspectives, this article offers a theory-generating analytic model to guide future empirical research, context-sensitive implementation, and policy deliberation. In doing so, it contributes to a more ethically grounded understanding of yoga as a potentially supportive, but conditional, wellbeing practice within displacement contexts, while cautioning against cultural essentialism, depoliticization, or claims of generalized effectiveness.

## Supplementary material

Supplementary Table S1 Final analytical corpus of 32 sources informing the structured narrative synthesis.
