# Supplementary material for: Yoga as salutogenic health promotion across displacement contexts: a culturally responsive public mental health synthesis
Source: Front Public Health. 2026 Jul 30;14:1815264. doi: 10.3389/fpubh.2026.1815264 (PMC13467834; doi:10.3389/fpubh.2026.1815264)
Supplement: Supplementary file 1 [file Table_1.docx]

**Supplementary Table S1. Sources included in the final analytical corpus**

This supplementary table lists the 32 sources comprising the final analytical corpus for the structured narrative synthesis.

| **First author and year** | **Study type** | **Topic or focus** | **Contribution to the conceptual model** |
| --- | --- | --- | --- |
| Antonovsky, 1979 | Conceptual/theoretical paper | Salutogenesis, stress and coping | Provides the foundational salutogenic paradigm, including the movement towards health and the role of general resistance resources. |
| Antonovsky, 1987 | Conceptual/theoretical paper | Sense of coherence | Defines comprehensibility, manageability and meaningfulness as the theoretical anchor for the coherence pathway. |
| Braun and Clarke, 2006 | Methodological paper | Thematic analysis | Supports the iterative thematic logic used to identify explanatory pathways and ethical boundary conditions. |
| Breedvelt et al., 2019 | Meta-analysis | Meditation, yoga, mindfulness, depression, anxiety and stress | Provides evidence on associations between contemplative and yoga-related practices and mental health outcomes, while supporting non-judgmental interpretation of heterogeneous interventions. |
| Cramer et al., 2013 | Systematic review/meta-analysis | Yoga and depression | Contributes evidence on yoga-related mental health outcomes and informs the discussion of symptom-based evidence and its limitations. |
| De Michelis, 2004 | Historical/theoretical paper | Modern yoga and transnational development | Supports the conceptualisation of yoga as heterogeneous rather than a singular intervention model. |
| Greenhalgh et al., 2018 | Methodological paper | Narrative review and evidence hierarchies | Supports the appropriateness of structured narrative synthesis for heterogeneous interdisciplinary evidence. |
| Herman, 1992 | Conceptual/theoretical paper | Trauma, recovery and safety | Informs the trauma-sensitive framing of bodily autonomy, pacing, safety and risks of distress amplification. |
| Inter-Agency Standing Committee, 2007 | Policy/guidance document | Mental health and psychosocial support in emergencies | Provides the layered-support framework used to position yoga as adjunctive rather than a substitute for protection, services or material assistance. |
| Jain, 2015 | Historical/theoretical paper | Yoga, commercialisation and popular culture | Supports caution against treating yoga as culturally neutral or universally meaningful. |
| Kirmayer et al., 2011 | Clinical guidance/review | Immigrant and refugee mental health in primary care | Informs the culturally responsive framing of care, explanatory models and refugee mental health practice. |
| Kleinman, 1980 | Conceptual/theoretical paper | Explanatory models of illness and care | Provides the anthropological basis for treating meanings of body, distress and care as locally mediated. |
| Koenig, 2012 | Review/conceptual paper | Religion, spirituality and health | Supports attention to the optional religious or spiritual meanings that may shape acceptability and interpretation. |
| Marmot, 2005 | Conceptual/review paper | Social determinants of health inequalities | Informs the structural and equity-oriented public health framing of psychosocial wellbeing. |
| Miller and Rasmussen, 2010 | Conceptual/review paper | War exposure, daily stressors and psychosocial frameworks | Provides a key displacement-informed account of daily stressors, chronic adversity and post-displacement conditions. |
| Pascoe and Bauer, 2015 | Systematic review | Yoga, stress measures and mood | Contributes evidence on stress-related outcomes relevant to the embodied self-regulation pathway. |
| Patel et al., 2018 | Commission/report | Global mental health and sustainable development | Frames public mental health through prevention, equity, social determinants and sustainable development. |
| Patton, 1999 | Methodological paper | Credibility in qualitative analysis | Supports the analytic emphasis on credibility, iterative interpretation and conceptual rigour. |
| Popay et al., 2006 | Methodological guidance | Narrative synthesis | Provides methodological guidance for structured narrative synthesis across diverse evidence types. |
| Porter and Haslam, 2005 | Meta-analysis | Predisplacement and postdisplacement factors in refugee mental health | Supports the displacement literature pathway by foregrounding social and contextual determinants of wellbeing. |
| Rogers and Pilgrim, 2014 | Conceptual/theoretical paper | Sociology of mental health and illness | Supports the critique of individualising distress and situates mental health within wider social and institutional conditions. |
| Sen, 1999 | Conceptual/theoretical paper | Capabilities, agency and development | Informs the framework's attention to agency, autonomy and structural conditions relevant to health promotion. |
| Silove et al., 2017 | Review/overview | Contemporary refugee mental health challenges | Supports the contextualisation of displacement-related mental health as shaped by conflict, uncertainty and social conditions. |
| Singleton, 2010 | Historical/theoretical paper | Modern postural yoga | Contributes to the conceptualisation of yoga as historically situated, diverse and contextually variable. |
| Sonnentag and Fritz, 2007 | Empirical/psychometric study | Recovery experiences: detachment, relaxation, mastery and control | Provides recovery-oriented constructs that may assist future operationalisation of salutogenic outcomes. |
| Steel et al., 2009 | Systematic review/meta-analysis | Mass conflict, displacement, torture and mental health | Supports the link between displacement exposure, post-displacement conditions and mental health outcomes. |
| Turrini et al., 2017 | Umbrella review | Common mental disorders in asylum seekers and refugees | Supports the displacement mental health evidence base and the need for cautious, context-sensitive implementation. |
| United Nations High Commissioner for Refugees, 2023 | Policy/statistical report | Global forced displacement trends | Provides the contemporary displacement context for the public mental health synthesis. |
| van der Kolk, 2014 | Conceptual/clinical text | Embodiment, trauma and recovery | Informs the cautionary discussion of embodied practice, trauma histories and potential adverse responses. |
| World Health Organization, 1986 | Policy/guidance document | Ottawa Charter for Health Promotion | Provides the health-promotion framework for enabling control over health and wellbeing. |
| World Health Organization, 2022 | Policy/report | World mental health and transformation of care | Supports the public mental health emphasis on prevention, equity and integrated systems of support. |
| Zaccaro et al., 2018 | Systematic review | Slow breathing and psychophysiological correlates | Supports the proposed mechanism linking breath regulation, autonomic modulation and perceived manageability. |
